# What Modeling Parasites, Transmission, and Resistance Can Teach Us

**DOI:** 10.1016/j.cvfa.2019.11.002

**Published:** 2020-03

**Authors:** Hannah Rose Vineer

**Affiliations:** Veterinary Parasitology, Department of Infection Biology, Institute of Infection and Global Health, University of Liverpool, Institute of Veterinary Science, Chester High Road, Neston CH64 7TE, UK

**Keywords:** Parasite, Ruminant, Modeling, Model, Climate change, Decision support, Anthelmintic resistance, Disease

## Abstract

Veterinarians and farmers must contend with the development of drug resistance and climate variability, which threaten the sustainability of current parasite control practices. Field trials evaluating competing strategies for controlling parasites while simultaneously slowing the development of resistance are time consuming and expensive. In contrast, modelling studies can rapidly explore a wide range of scenarios and have generated an array of decision support tools for veterinarians and farmers such as real-time weather-dependent infection risk alerts. Models have also been valuable for predicting the development of anthelmintic resistance, evaluating the sustainability of current parasite control practices and promoting the responsible use of novel anthelmintics.

## Key points

• Models are valuable for exploring complex parasite systems, especially when field trials would be costly or impossible.
• Research and development of novel approaches to parasite control can be model-guided, for example, vaccine development.
• Optimal control strategies can vary based on prevailing environmental conditions resulting from the impact of weather on the abundance of parasites, and modeling provides a means to compare the success of different strategies under such varying conditions.
• An array of model-based decision support tools is available for veterinary clinicians and farmers to facilitate sustainable parasite control practices.
• Modeling can provide evidence to guide and support policy on the sustainable control of parasites, especially the responsible use of new anthelmintics.

## Introduction

Antiparasiticide resistance is widely reported in a range of ectoparasites and endoparasites^1^^,^^2^ and is set against a backdrop of environmental change. Climate warming may have already changed the geographic distribution and seasonal abundance of some parasites,^3^ and interannual climate variability could result in unexpected differences in the seasonal risk of parasitic infection between years.^4^ These factors may also affect the local relevance of field studies performed decades ago before large-scale environmental changes.

Strategies that advocate more thoughtful and targeted applications of antiparasiticides promise to slow the development of resistance^2^^,^^5^ while nonchemotherapeutic approaches offer promising alternatives.^2^^,^^6^^,^^7^ However, host-parasite dynamics are complex, especially because of the diversity of ruminant livestock production systems used worldwide. Capturing this variability sufficiently using field trials alone would be prohibitively expensive and likely impossible. Furthermore, field trials can only be undertaken in the environmental conditions encountered during the trial and cannot capture interannual variability in weather patterns nor potential future climate change. Models offer an additional tool to complement and drive forward the development of novel approaches to parasite control, to further the understanding of host-parasite and epidemiologic processes and the development of drug resistance, and to generate decision support tools for veterinarians and farmers.

This article aims to provide veterinary practitioners with an understanding of what models are as well as their advantages and potential limitations, signpost model-based resources for assessing parasite disease and transmission risk, and highlight key areas where models are helping to shape the development of sustainable parasite control strategies.

## Wrong, but useful

### What Are Models, and Why Do We Use Them?

**Fig. 1:**
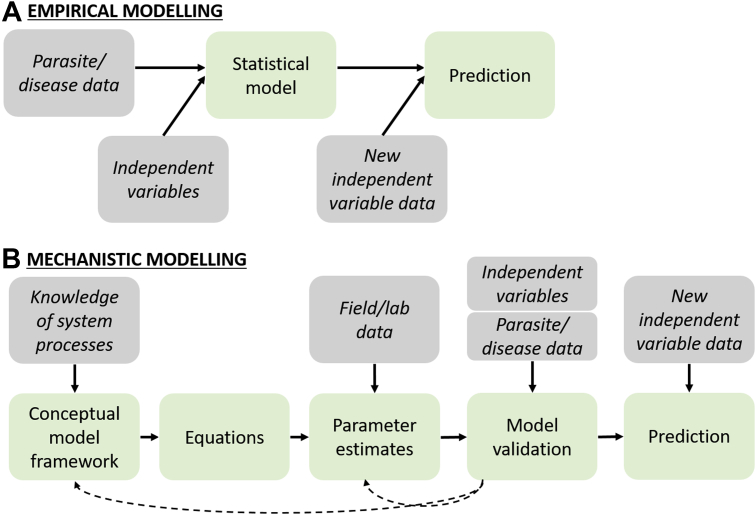
Comparison of typical empirical (*A*) and mechanistic (*B*) modeling processes. Data inputs are shown in grey boxes (italicized font if viewing in grayscale) and key steps in the modeling process are shown in green boxes. The processes are described in detail in Box 1.

Models are simplified, mathematical representations of real-world systems or events that either broadly describe the relationship between a variable of interest and a predictor variable (empirical model; Box 1 and see Fig. 1A), or describe the system processes as a set of mathematical equations (mechanistic model; see Box 1 and Fig. 1B).

Box 1 **Types of models**

Models can be broadly described as either empirical or mechanistic. To understand the differences between these models, it is useful to first understand the model development process (Fig. 1).

**Empirical modeling**

The empirical modeling process (see Fig. 1A) typically involves relating parasite or disease data to independent variables (such as temperature, vegetation indices, farm characteristics, and parasite control strategies) using statistical models, to explain epidemiologic patterns or species’ spatial distributions. The resulting model can be used to make predictions based on new independent variable data. Very little knowledge or understanding of the processes underlying the relationships is needed; therefore, the process is less data intensive than mechanistic modeling. However, care needs to be taken extrapolating the findings beyond the range of the data used to develop the model, because correlations between independent variables may change in time and space.

*Empirical modeling example.* Bryan and Kerr^8^ developed an empirical model predicting gastrointestinal (GI) nematode larvae density on pasture (ie, an indirect measure of transmission risk) by relating monthly measures of larvae recovered from pasture in Queensland, Australia between 1975 and 1979, to temperature, rainfall, and dung beetle activity using a regression model. The model predicted that rainfall increased larval recovery from pasture while dung beetle activity reduced larval recovery. Based on the model, the authors made predictions to inform the optimal timing of anthelmintic treatments: they predicted that larval recovery would increase 92% following a 100-mm increase in rainfall, that beetle damage to pats could result in a reduction of between 57% and 94%, and therefore that treatments are best applied during the winter months when rainfall is high and beetles are inactive to reduce transmission risk. However, the effects of temperature could not be separated from the effects of dung beetles and rainfall, and the model may not be applicable to other regions nor beyond the late 1970s.

**Mechanistic modeling**

The mechanistic modeling process (see Fig. 1B) requires a detailed understanding of the processes underlying the epidemiology of parasite/disease dynamics to develop a conceptual framework (simplified representation of the processes) and mathematical equations representing the system processes, such as parasite establishment in the host. Model parameters such as death and transmission rates are then estimated using laboratory and field data. The models are usually validated using relevant independent variable data and parasite/disease data. If validation identifies significant discrepancies between the model predictions and these data, the model framework and parameter estimates are revisited and improved. Finally, the validated model can be applied to new independent variable data to make predictions. Mechanistic model development is usually much more data intensive than empirical model development, and as a result it is difficult to develop models for systems in which limited data exist (eg, understudied parasite species). However, as mechanistic models incorporate system processes and make fewer assumptions about correlations between independent variables, they are useful for projecting onto new conditions such as climate change.

**Fig. 2:**
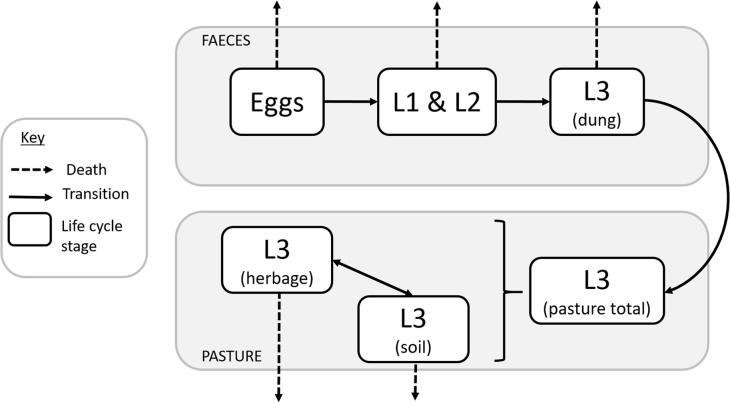
Mechanistic model development is aided by conceptual frameworks, which visualize the current understanding of the system, help formulate mathematical equations, and identify key parameters. This conceptual framework details a model developed for the population dynamics of trichostrongylid gastrointestinal nematodes infecting ruminants.^9^ Based on previous research, it is known that eggs are deposited in dung and develop (transition) through 2 larval stages (L1 ad L2) to reach the third, infective, larval stage (L3). L3 then migrate (transition) out of the dung onto pasture, where the total L3 on pasture is partitioned between the soil and the herbage. Data in the literature were available to estimate death rates for each life-cycle stage, development rates from egg to L3, and bidirectional migration between the soil and herbage, based on temperature. Further controlled observations were required to estimate the influence of moisture on the rate of migration between dung and pasture. Because trichostrongylid nematodes share the same life cycle, the model can be adapted for different species by simply adapting the death and transition rates.

For example, Rose and colleagues^9^ developed a mechanistic model framework for the development, survival, and migration of ruminant gastrointestinal nematodes on pasture, based on current understanding of the life cycle and behavior of trichostrongylid nematodes (Fig. 2). Model parameters (death rates and transition rates such as development and migration) were estimated based on data in the literature (eg, the survival and development of eggs and larvae incubated in dung), and additional controlled field observations of larval migration from dung in response to rainfall. The model was validated using pasture larvae counts from a commercial farm and additional independent data sets from the literature. Adaptations of this model have been applied in a range of scenarios, for example to predict the potential epidemiologic benefits of breeding nematode-resistant ewes under climate-change scenarios,^10^ and similar models have been used to identify optimal treatment strategies to delay the development of anthelmintic resistance.^11^

While the process of developing a model can enhance understanding of parasites and epidemiologic processes, and focus attention onto specific topics for future research, models also allow rapid exploration of the impacts of change (eg, climate change^9^) or interventions (eg, the timing of antiparasiticide treatments^11^). To achieve this, usually the model input, such as climatic data representing different regions or time periods, or the values assigned to model components (parameters), are adjusted to represent the different scenarios of interest. Testing each of the scenarios in the “real world” would require a different field trial or controlled experiment, which can be prohibitively costly. In addition, the development of resistance to antiparasitic drugs can take years if not decades, so running field trials to compare the impact of different treatment strategies is challenging. The impacts of regional differences in climate on parasites and disease can also only feasibly be tested in the field at a limited number of locations and only over short time scales, thus usually representing a limited range of weather patterns over a 1- to 5-year period. It is also impossible to truly test parasites’ responses to climate change in the field, as these conditions are not yet realized, or to monitor phenomena that are rare at present and cannot be measured, such as development of resistance to novel antiparasitic compounds. By contrast, models can rapidly explore large numbers of hypothetical scenarios and can be projected onto weather data for multiple regions or future climates, providing insight into parasite epidemiology that would otherwise be inaccessible without significant time and funding. As a result, models are applied in all fields of veterinary parasitology, including the management of drug resistance,^11^ generating climate impact assessments,^9^ informing vaccine development,^12^ and informing selective breeding for nematode resistance.^10^

### Model Uncertainty and Validation

Since all models are wrong the scientist must be alert to what is importantly wrong.

—George Box (statistician), 1976^13^

George Box’s words, which are widely paraphrased as “All models are wrong, but some are useful,” highlight a fact that is easy to overlook: models will always produce predictions and output that are to some extent uncertain. They are imperfect representations of real-world observations, which themselves are imperfectly measured. How accurately a model represents the study system, and therefore how useful a model is likely to be, can be assessed by model validation. Usually this is done by comparing model output with field observations (see Box 1). Even fairly complex models can be validated through empirical testing of key findings, for example by designing field trials to specifically test model outcomes.^14^^,^^15^

The aim is not to perfectly reproduce the host-parasite system but to produce models that are useful; whereas it is impossible to eliminate uncertainty and avoid making assumptions, it is possible to produce useful models that replicate the system of interest in sufficient detail, or provide opportunities to compare scenarios. For example, the rate of development of anthelmintic resistance is difficult to measure in the field because it typically occurs over a period of years and is imprecisely measured using currently available technology. In this context, models provide an opportunity evaluate the relative impact of control strategies that may enhance or delay the development of resistance over extended time periods.^11^

### Is Complex Always Better?

This then begs the question: are complex models always better? If 2 competing models produce equally useful and accurate output, the simplest model is preferable.^13^ However, there are instances when additional complexity is beneficial and, in general, mechanistic models are preferred over empirical models if sufficient data are available for model development. The key requirement is that the models, as accurately as possible, represent the biology they are attempting to reproduce.

**Fig. 3:**
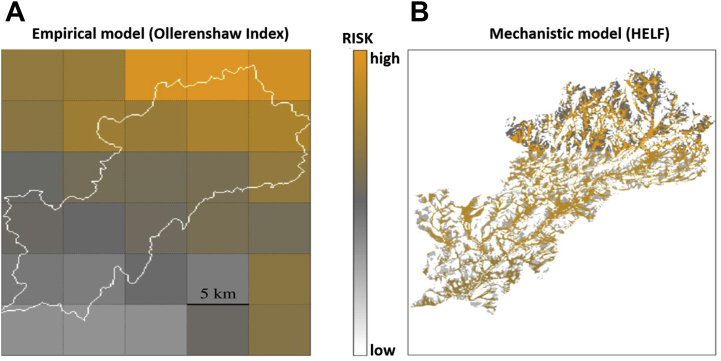
One of the factors limiting the application of models developed to inform parasite control strategies for ruminant livestock is the availability of the data that can be used as model input. For example, weather stations may be some distance from the farm, and gridded weather data are often low resolution (eg, several km^2^) in comparison with the scale at which transmission takes place. Comparing output of an empirical model (“Ollerenshaw Index”^16^; *A*) and a mechanistic model (“HELF: Hydro-Epidemiological Liver Fluke model”^18^; *B*) for risk of *F hepatica* infection, for a river catchment area in Wales, UK, demonstrates how mechanistic modeling may provide a solution. High risk of infection is shown in orange, moderate risk is shown in gray, and low risk is shown in white. The empirical model output (*A*) may be useful to highlight larger regions at high risk of fasciolosis caused by high rainfall. However, a moderate to high risk was predicted throughout the catchment despite the fact that spatial risk often varies between and within fields owing to heterogeneity in suitable habitats for the snail intermediate host, which is determined in part by hydrologic processes. Beltrame and colleagues^18^ coupled a mechanistic model of hydrologic processes with a simple mechanistic model of the population dynamics of *F hepatica* to predict metacercariae abundance depending on rainfall runoff and soil moisture (*B*; high abundance is shown as high risk). This mechanistic model used the same low-resolution weather data as the empirical model (*left*) but was able to predict risk at a finer spatial scale (25 m) by coupling this with high-resolution topography (elevation) data. The model predicted that much of the area predicted to be moderate to high risk using the empirical Ollerenshaw index (*A*) was actually likely to be low risk (*white*, *right*). These results could be used to plan grazing strategies to avoid infection.

Different approaches to modeling *Fasciola hepatica* (liver fluke) risk illustrate this point. Empirical models have been developed since the 1950s to predict the risk of *F hepatica* infection by relating the incidence of fasciolosis (or measures of exposure) to environmental conditions. Although empirical models are useful to predict the risk of fasciolosis over time and space within the region where they were developed, care must be taken when extrapolating outside of these regions and into future climatic conditions because model accuracy under “new” environmental conditions is unknown. For example, Ollerenshaw and Rowlands^16^ developed a model for risk of fasciolosis in Anglesey, United Kingdom. Because of its simplicity there is considerable potential for this model to be widely used to predict the seasonal risk of fasciolosis. However, the model was developed for the specific environmental conditions in Anglesey at the time of model development (temperate, with high rainfall year-round), and further validation would be necessary to assess its accuracy when applied outside of Anglesey or using current and future climatic data. Furthermore, the risk of parasite infection often varies at finer spatial scales than the available climatic data (eg, parasites clustered within a farm or field,^17^ compared with several-km^2^ resolution of climatic and weather data). This potentially limits the application of empirical models as decision support tools for farmers and veterinarians. In the case of *F hepatica*, mechanistic models^18^ offer a solution to both of these limitations by explicitly modeling the processes underlying the relationships between the host, parasite, and environment (Fig. 3).^16^^,^^18^

(*Adapted from* Beltrame L, Dunne T, Rose Vineer H, et al. A mechanistic hydro-epidemiological model of liver fluke risk. J Roy Soc Interface. 2018; 15(145).)

The implications of this are that veterinarians and their clients should ensure they are aware of the potential limitations of models so as to appreciate the uncertainty in model predictions. Model output should be interpreted in the context of farm management and local variation in weather/microclimate if the models are to be used to guide parasite control choices (used as decision support tools). For example, *Nematodirus battus* egg hatch predictions^4^ based on a network of weather stations are provided in a Web-based tool alongside “rules of thumb” that can be used to adjust for local microclimate (aspect of fields and height above sea level) and farm management to assess risk (Table 1).

**Table 1:** Ruminant parasite decision support systems implementing models

DSS	Region	URL	Description	Model
Ask Bill	Australia	www.askbill.com.au	Predicts sheep well-being and productivity based on weather and farm management. Incorporates models of gastrointestinal nematode populations and blowfly strike risk	Kahn et al,^19^ 2017
eggCounts	Global	http://shiny.math.uzh.ch/user/furrer/shinyas/shiny-eggCounts/	User interface to apply advanced analysis to fecal egg count and fecal egg count reduction test data	Wang et al,^20^ 2017
Flyboss	Australia	www.flyboss.com.au	Predicts risk of blowfly strike to optimize treatment timing and compares multiple management options	Horton & Hogan,^21^ 2010
LiceBoss	Australia	www.liceboss.com.au	Predicts the probability of infestation of sheep by *Bovicola ovis*, and the level of wool damage caused, to inform treatment decisions	Lucas & Horton,^22^ 2014; Horton et al,^23^ 2009
NADIS Blowfly alerts	UK	www.nadis.org.uk	Predicts *Lucilia sericata* abundance based on recent weather data	Wall et al,^24^ 2000
Paracalc	Global	www.paracalc.be	Predicts the economic impact of nematode, liver fluke, and sheep scab infections in cattle, simulates the impact of treatment strategies on gastrointestinal nematodes, and provides decision support for liver fluke control	Charlier et al,^25^ 2012
SCOPS *Nematodirus* alerts	UK	www.scops.org.uk	Predicts the timing of *Nematodirus battus* mass hatch in spring, depending on daily temperature data	Gethings et al,^4^ 2015

URLs correct at June 14 2019.

*Abbreviations:* DSS, decision support systems; SCOPS, sustainable control of parasites in sheep.

## Contribution of modeling to advances in ruminant parasitology

Models of ruminant parasites and parasite transmission span several decades for both endoparasites^26^ and ectoparasites.^24^^,^^27^ These modeling efforts have contributed to several advances in ruminant parasitology, broadly categorized as advances that enhance the scientific understanding of epidemiology and disease processes, and advances that are of practical benefit to enhance parasite control capabilities.

Mechanistic models are indispensable as tools for testing and broadening epidemiologic understanding of host-parasite systems, driving forward research. For example, they have been used to identify the level of protection required from *F hepatica* vaccine candidates^12^ and aid comprehension of endemic stability in bovine babesiosis, a complex epidemiologic process that depends on the balance of tick numbers, pathogen prevalence, and age structure of the host population.^27^

Beyond broader scientific advances, there is now a plethora of models producing clinically relevant output, particularly to support and encourage more sustainable approaches to parasite control.

### Reducing Reliance on Veterinary Medicines

Targeted treatment is a key aspect of sustainable parasite control, ensuring antiparasitic treatments are applied at the right time while avoiding unnecessary treatments.^2^ Related to this is the potential to avoid infection by moving livestock away from high-risk pasture or sources of infection. However, the timing of peak risk of infection often depends on climate (or recent weather patterns) and farm management,^24^ and the optimal timing of treatment or other interventions (such as grazing movements) depends on these factors as well as economic considerations. Since these factors are difficult for veterinarians and farmers to track, and it is difficult for a nonexpert to relate this to parasite risk because of the complexity of the host-parasite-environment system, models can play a key role in providing decision support (see Table 1).

### Minimizing Selection for Anthelmintic Resistance

**Fig. 4:**
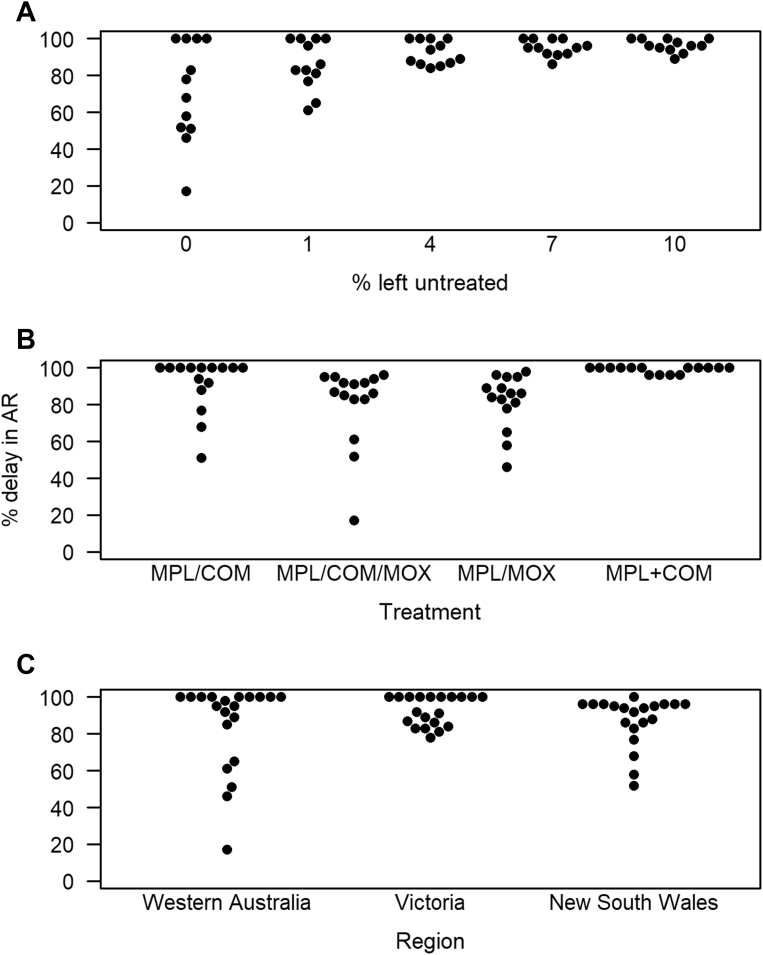
Using models it is possible to simulate processes that are not easily measurable in the field (such as the development of anthelmintic resistance [AR]) over extended time scales. For example, Dobson and colleagues^33^ simulated the population dynamics of multiple trichostrongylid nematode species infecting sheep in Australia, and the development of AR in these populations in response to a range of treatment strategies. The efficacy of each strategy was expressed as a percentage delay in the development of AR over a 20-year period compared with control scenarios whereby flocks were left untreated or treated exclusively with monepantel (MPL), moxidectin (MOX), or a combination (COM). Simulations varied the percentage of adult stock left untreated and the anthelmintic products used, and were replicated using weather data from 3 regions in Australia. Data shown were extracted from Tables S2–S4 of the original publication.^33^ Points represent the output of model simulations. Simulations suggest that leaving even a small proportion of the flock untreated delays the development of resistance (*A*). However, how effective this strategy is in delaying AR was variable (eg, leaving 1% untreated results in approximately 60%–100% delay in AR; (*A*), depending on the treatments used (*B*) and regional weather/climatic conditions (*C*). Crucially, with the exception of MPL + COM combination treatment, which was always 96% to 100% effective, the optimal treatment strategy varied by region, highlighting the importance of considering environmental conditions in the development of sustainable parasite control strategies. −, treatments applied in rotation; +, treatments applied in combination; COM, combination treatment of benzimidazoles + imidazothiazoles + abamectin; MOX, moxidectin; MPL, monepantel.

Along with decision support tools, models of anthelmintic resistance in gastrointestinal (GI) nematodes have been hailed as one of the 10 events defining anthelmintic research.^28^ Models of anthelmintic resistance (Table 2) usually track GI nematode populations over time, dividing the population according to the genotype (eg, RR for homozygotic-resistant nematodes, RS for heterozygotic-resistant nematodes, and SS for homozygotic-susceptible nematodes). A different proportion of nematodes of each genotype are removed from the population when anthelmintic treatments are applied (eg, removing all or most of the susceptible nematodes, none or very few of the resistant nematodes, and an intermediate number of the heterozygotic nematodes). These models are valuable for comparing parasite control strategies and to identify methods that minimize selection for resistance^33^ (Fig. 4).

**Table 2:** Selected examples of model evaluation of sustainable parasite control practices

Parasite Control Strategy	Enhances (+) or Slows (−) Anthelmintic Resistance	Model
Treating ewes at lambing	+	Leathwick et al,^29^ 1995; Leathwick et al,^30^ 1997
Treating ewes in autumn	+	Leathwick et al,^29^ 1995
Increasing treatment frequency	+	Leathwick et al,^29^ 1995
Moving hosts to “clean” grazing (dose & move)	+	Leathwick et al,^29^ 1995
Set stocking	+	Barnes & Dobson,^31^ 1990
Grazing untreated ewes with treated lambs on same land after weaning (ewes follow lambs)	−	Leathwick et al,^29^ 1995; Leathwick,^32^ 2012
Targeted selective treatment/Leaving a proportion of hosts untreated	−	Dobson et al,^33^ 2011; Berk et al,^34^ 2016
Single mid to late grazing season treatment with novel anthelmintic	−	Leathwick & Hosking,^11^ 2009
Rotating anthelmintic classes annually	−	Learmount et al,^35^ 2012
Persistent anthelmintics (includes long-acting and controlled-release devices)	+/−	Le Jambre et al,^36^ 1999; Barnes & Dobson,^31^ 1990; Leathwick et al,^30^ 1997; Dobson et al,^37^ 1996
Combination anthelmintics	+/−	Leathwick et al,^14^ 2012; Learmount et al,^35^ 2012; Leathwick,^32^ 2012
Weather/climate	+/−	Dobson et al,^33^ 2011

Whether the parasite control strategy is predicted to enhance or slow the development of anthelmintic resistance is shown as + or −, respectively. Predictions that vary by study or vary depending on interacting factors are shown as +/−.

Models of anthelmintic resistance have highlighted unsustainable parasite control practices, such as anthelmintic treatment of ewes at lambing^29^ (see Table 2), which have later been confirmed by field data.^15^ They have also demonstrated the considerable potential for sustainable parasite control guidelines^38^ to slow the development of resistance.^35^ High-risk practices identified by modeling studies (see plus signs in Table 2) could be avoided to minimize selection for resistance. If they cannot be avoided, practices that are predicted to slow the development of resistance could be implemented to help mitigate the impact of the high-risk practices (see minus signs in Table 2).

The responsible and strategic use of new anthelmintics (also known as “new actives”) is of paramount importance. Just 1 year after the discovery of aminoacetonitrile derivatives (monepantel) was published, a modeling study provided evidence that a single annual treatment with a novel anthelmintic could slow the development of resistance to other, older anthelmintics, especially when applied later in the grazing season.^11^ The use of novel anthelmintics in lambs in the mid-to-late grazing season is now advocated.^38^ Field trials to test this would have taken years to complete, at significant cost.

Two independent modeling studies subsequently simulated the development of resistance to novel anthelmintics such as monepantel and derquantel, predicting that over a period of 40 years the rate that anthelmintic resistance develops to a novel compound could be slowed when the novel anthelmintic is administered as a combination with another anthelmintic class.^32^^,^^35^ These studies were notable because they were completed before the first report of detectable anthelmintic resistance to monepantel, at a time when field studies to track the development of resistance to novel anthelmintics would have been impossible.

Veterinarians and policymakers should also consider potential interactions that may or may not be included in modeling studies. For example, Le Jambre and colleagues^36^ predicted that the use of persistent anthelmintics in lambs (controlled-release ivermectin or persistent moxidectin oral drench) would lead to rapidly developing anthelmintic resistance (compared with nonpersistent ivermectin oral drench). The investigators concluded that “treating sheep with a persistent ML [macrocyclic lactone] while grazing on a contaminated paddock should be seen as an emergency procedure when there are no alternatives.” Similarly, other modeling studies predict that the magnitude of the impact of persistent anthelmintics on the development of resistance varies depending on grazing management.^31^

These examples highlight a central theme to managing and slowing the development of resistance: the size of the population of nematodes that are in *refugia*, having not been exposed to anthelmintic treatment. The impact of weather and climate on the abundance of parasites and seasonal dynamics of parasite populations (and thus the size of the *refugia* on pasture) is of increasing interest in the veterinary parasitology research community (eg, Verschave and colleagues^26^). Modeling studies exploring the interacting effects of climate and farm management on the development of anthelmintic resistance are limited to date. However, Dobson and colleagues^33^ predicted that the optimum anthelmintic treatment strategy varied by Australian region and the resulting differences in the size of the *refugia* on pasture (see Fig. 4). Further model development is ongoing to evaluate the impact of climate and climate-management interactions on the development of anthelmintic resistance, and recent progress has been made with similar models evaluating the impacts of climate-based and *refugia*-based control strategies on the development of anthelmintic resistance in equine cyathostomins,^39^^,^^40^ paving the way for similar developments in ruminant parasitology.

## Summary

Models of parasites, their transmission, and the evolution of anthelmintic resistance have made significant contributions to veterinary parasitology in recent decades. Most recently, models have provided evidence to guide the responsible use of novel anthelmintics at a time when field trials to optimize the timing and method of administration to minimize selection for resistance would have been impossible. In parallel with the threat of developing drug resistance, ruminant producers must contend with increasingly variable weather patterns and the threat of climate change, which affects parasite abundance and risk of infection. An array of decision support tools are now available to farmers and veterinarians to help plan and implement targeted parasite control strategies that are tailored to the prevailing weather conditions. The examples presented throughout this article highlight how modeling is an indispensable tool in veterinary parasitology.
